# Expanding knowledge and roles for authority and practice boundaries of Emergency Department nurses: a grounded theory study

**DOI:** 10.1080/17482631.2018.1563429

**Published:** 2019-02-14

**Authors:** Yanny Trisyani, Carol Windsor

**Affiliations:** a Emergency Nursing and Critical Care Nursing Department, Universitas Padjadjaran, Bandung, Indonesia; b Postgraduate Research Coordinator, Queensland University of Technology, Brisbane, Australia

**Keywords:** Authority, Emergency Department (ED), emergency nursing, expanding knowledge, nursing roles, grounded theory (GT), practice boundaries, self-regulation

## Abstract

**Purpose:** While emergency department nurses in Indonesia are critical to quality care, the role lacks recognition and standard practices and regulation of scope of practice are absent. This research explored the role of nurses in Indonesian EDs.

**Method:** The conceptual lens applied in the research was grounded theory. The main data source was 51 semi- structured interviews with 43 ED nurses, three directors of nursing, three nurse leaders and two nurse educators. Data were also generated through observations and memos.

**Results:** Two key categories were constructed; *shifting work boundaries and lack of authority*. Shifting work boundaries was symbolic of a lack of professional authority and legitimized knowledge. Lack of authority reflected the dimension of professional autonomy through the nexus of power and knowledge. The interrelationship of these two concepts constructed a core category, *securing legitimate power*, which underpinned the positioning of nursing in Indonesia.

**Conclusions:** The interconnection between political gains, tertiary knowledge, professional regulation and implementation of gender-sensitive policies was critical to the development of the ED role, the positioning of nursing within the health care system and improvement in quality of care.

## Background

Increasing demand for emergency care services in Indonesia is linked to social economic change and demographic factors. The demographic factors are indicative of the pressure on the Indonesian health care system, with a population of close to 264 million in 2017 (UNDESA, 2017). Indonesia is the world’s fourth largest country in terms of population size (World Bank Group, 2017). The density of the population has created huge demands on the existing health care system.

**Figure 1.**
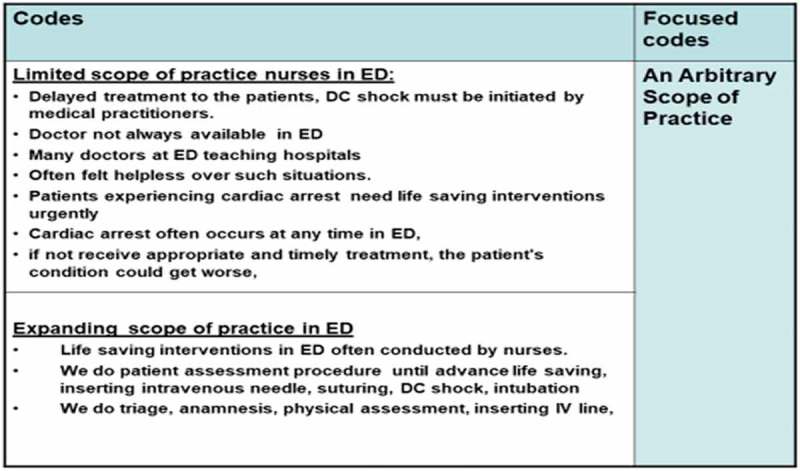
Example of focused coding procedures.

In addition, social economic and industrial growth and associated lifestyle transformations have broadened the sphere of activity of health professionals, including the Emergency Department (ED) nurse. Indeed, the high numbers and high complexity of patients presenting to Indonesian EDs is linked to the rapid growth of the urban population and increased intercity traffic and shifting lifestyle. The five leading causes of death in adults in Indonesia, recorded in 2014, were cardiovascular diseases (37%), cancer (13%), injury (7%), diabetes (6%), and chronic respiratory diseases (5%) (World Health Organization, 2014). These burdens of illness reflect a high incidence of severe cases that are related to high demand for emergency care and high numbers of patients presenting to Indonesian EDs. The demographic and social factors and Indonesian environmental conditions, which include a high risk of natural disasters, impose enormous demands on the country’s health care services and emergency services.

As a major front-line service for emergency response intervention, the ED is increasingly under pressure to provide quality health care. Nurses at the front line in daily practice in the ED hold a strategic position in the provision of timely and safe emergency care services to society.

Yet, in Indonesia, emergency nursing practice is still characterized by the absence of standard practices and competencies; scope of practice is also unregulated, as is the case for all nursing specialities in this country (Suba & Scruth, 2015). Lack of nursing regulation and minimum standards for practice reflect the inequitable position of nursing within the Indonesian health care system. Indonesian nursing law was approved in 2014, following a 25-year struggle. Nursing law reflects the rights to self-regulate and to shape nursing practice (Adams, 2009); it is therefore critical for autonomy and professional development of nursing (Coburn, 2006). The lack of nursing regulation has led to the less powerful voice of nursing in health care. “*Nursing still does not have a proper position in that decision-making process*” (Indonesian National Nurses Association [INNA], 2011). The absence of a voice has impacted the capacity of ED nurses in the clinical setting because the lack of political support has constrained the ED nursing role.

Internationally, emergency nursing work involves specialized ED nursing roles. Nursing activities as front-line care, which have characterized the ED nursing role, are defined as resuscitation and advanced life support (Ena, 2012), management of life-threatening injury such as trauma (Fry, 2011a), and triage (Fry & Stainton, 2005). The ED nursing role in triage processing has been found to have produced a dramatic reduction in waiting times (Bruijns, Wallis, & Burch, 2008), and it has significantly reduced the percentage of patients who leave EDs without being seen (Love, Murphy, Lietz, & Jordan, 2012). A further important role of senior ED nurses is in patient management (Fry, 2011a) and particularly of severe traumatic brain injury (Damkliang, Considine, Kent, & Street, 2014). Nursing work in an ED is also involved in non-clinical dimensions of organizational and interdepartmental management (Nugus & Forero, 2011). Nursing work in an ED has also extended to the provision of health care services to the wider society in the event of disaster (Hammad, Arbon, Gebbie, & Hutton, 2012; Ranse et al., 2013; Usher et al., 2014) and in special cases such as forensic nursing (Çalışkan, Karadağ, Yıldırım, & Bingöl, 2014; Pasqualone, 2015). All of these findings give support to the link between the ED nursing role and advanced knowledge and skills at the speciality level that underpin the nursing role. The emergency nursing role in speciality practice is deemed crucial for both improving the quality of emergency care services to the society and for nursing as a profession.

However, a lack of role clarity in relation to advanced practice nursing roles and poorly defined responsibilities still exist. There is lack of certification examinations and variance in the level of state recognition in advanced practice nursing (Glover’s, 2006) and variations in definitions of the advanced practice role and associated responsibilities (Fry et al., 2013). Variations still exist in terms of the substance of the roles, their titles and in the educational backgrounds at higher education that currently support advanced nursing roles.

The implementation of advanced practice nursing roles has been found to contribute to reductions in patient waiting time, improved quality of care, increased patient and staff satisfaction, and to the retention of senior nurses in ED settings (Hudson & Marshall, 2008; Melby, Gillespie, & Martin, 2011), and it has improved community access to emergency care (Carter & Chochinov, 2007). Nonetheless, advanced nursing roles in the area of emergency nursing have received professional and social recognition in Western societies. However, in Indonesia formal recognition in advanced nursing roles in emergency nursing areas has not yet been implemented.

An understanding of the nursing role in the ED setting also means recognizing the position of nursing in the Indonesian health care context, which is embedded in a broader social system. Thus, the lack of clarity around the ED nursing role is related to the social position of nursing in Indonesia. This situation has undermined the position of nursing in Indonesia and impacted on the capacity of ED nurses to respond effectively to critically ill and unstable patients.

In light of the apparent gap in knowledge regarding the nexus of advanced knowledge, authority, and nursing at the clinical and macro national level, an exploration of the role of ED nurses within the Indonesian context was thus significant. It also has implications for improving the quality of emergency care services and understanding the position of nursing at both the clinical ED and Indonesian health care levels. Generally, this study is significant for understanding the interrelationship of advanced knowledge, power, or authority and nursing, which is critical for positioning nursing in the health care context and the wider social system.

## Methods

### Aims

The aims of this research were to explore the role of ED nurses working in general hospitals within the Indonesian context and the interrelationship of advanced knowledge, power, or authority and nursing, which is critical for positioning nursing in health care context and the wider social system.

### Design

The constructivist grounded theory of Charmaz was employed as the methodological approach for this study. GT was initially conceptualized as a set of research methods, the aim of which was to construct theoretical explanation from data, using comparative methods, coding procedures, and interpretations as the main analytical strategy. Charmaz’s (2005, 2009, 2014) constructivist GT is appropriate to this research, where meaning and knowledge are understood as socially produced through social practices and social interaction. The concept of “social” is important because it means that structures and actions cannot be conceived as disconnected. In other words, structure cannot be understood apart from actions, but at the same time, actions are conditioned by structure. Meaning and knowledge, therefore, are both shaped within cultural, historical, social, and structural contexts, and are temporal in nature (Charmaz, 2009). In this research, the GT standpoint assumes that the nursing role is embedded in a larger social process related to cultural, historical, political, and other significant factors that exist within the context of the research. This research approached the participants as individual social actors and meaning and knowledge are negotiated and shared socially. Thus, data in this research were considered mutually constructed by the researcher and participants.

### Research setting and participants

The research was conducted in three EDs at the three general hospitals located in three cities in West Java, Indonesia. Permission to undertake this research was obtained from the three directors of the respective hospitals. Ethics approval was acquired from the Hospital Ethics Committee and from the QUT-University Research Ethics Committee in Australia.

In accessing the ED participants, informal permission from the heads of ED and directors of nursing at the hospitals was also obtained. The nurse managers in the three EDs were the first people to be approached by the researcher in those ED settings. The researcher invited participation through a display of posters in the EDs and the dissemination of information by nurse managers at each ED.

The principal investigator has working experience as an emergency nurse. The researcher was aware that some ED nurse participants might know the researcher as a result of shared clinical experience and so she made an effort to explain her position as a researcher at the beginning of the participant’s recruitment process.

All participants were provided with both a verbal and a written description of the study aim, their involvement, and the time commitment. Assurances of anonymity, confidentiality, and the participants’ rights were also provided. All participants provided their written informed consent prior to the commencement of interview.

Participants in the data collection phase, *phase one*, were recruited purposively according to the following criteria: nurses who had been working at least 3 years in an ED setting (this enabled the participants to have rich information); willing to share their experiences and to participate in audio recorded interviews; willing to be observed in their own environment. In *phase one*, 35 participants were recruited from the selected EDs. In *the theoretical sampling phase*, 16 further participants were recruited and the total sample constituted 51 nurses who contributed to a full exploration of the phenomenon under research.

### Data collection

The processes of data collection and analysis were undertaken simultaneously and were conducted as follows: contextual observations; participant interviews; and theoretical sampling interviews.

The researcher invited participation through a display of posters in the EDs. Verbal and written information regarding the study was provided. All participants provided their written informed consent prior to the commencement of data collection.

The data collection phase was conducted through contextual observations and a series of face to face participant interviews.

Contextual observation was undertaken in each ED for around 6 hours as an initial step in the data collection process and was conducted prior to participant interview. The intention was to gain a global understanding of the ED environment or the context of this research. Observational data were recorded in field notes and researcher memos. Conceptual insights generated from the observational data, or what Glaser (1978) terms theoretical sensitivity, informed the framework for the participant interviews.

Participant interviews were the main data source for this study. The interviews involved 35 ED nurses and were organized in two phases: initial interviews and a second round of interviews.

The initial interviews involved individual semi-structured interviews with 10 ED nurses (4 nurses from ED A; 3 nurses from ED B; and 3 nurses from ED C). The interviews were performed in the Indonesian language and audio-recorded. Each interview commenced with open-ended questions:

How do you work as a nurse in emergency care services; describe and define your role?

Further questions were posed for the purposes of clarifying participant responses, or based on what had been viewed during observation. Furthermore, focused questions sought to explore more deeply and in greater detail the significant issues expressed by participants. This interview process allowed for the expression of feelings, views, thoughts, and actions (Charmaz, 2014), to reflect upon their experiences of the ED nurses role.

The first interview was transcribed in Indonesian and initial coding commenced in that language. Significant analytical concepts produced from the first interview were further explored in a second participant interview. Thus in the initial interviews simultaneous processes of interview and analysis were carried out sequentially. The duration of the interviews was on average 50–70 minutes. Focused coding was conducted following initial coding. Significant issues generated at this stage were further explored in the second interviews.

The second round of interviews encompassed 25 ED nurse participants and it were conducted sequentially in the EDs of hospital A with 11, hospital B with eight, and hospital C six participants. In this phase, the interview questions were shaped by significant issues produced through the focused coding process of initial interviews.

Thus the first phase involved 18 hours of observations and 35 participant interviews consisting of initial interviews with 10 ED nurses and following some analysis further interviews with 25 participants.

The second phase data collection was *t* *heoretical sampling*, which involved interviews with 16 participants. These interviews involved five ED nurses, three ED nurse managers, three Directors of Nursing, three nurse leaders at the national level, and two nurse educators. The purpose of these interviews was to fill analytical gaps found in the previous analysis phase, and to illuminate and refine emergent concepts. Following this, all other data sources, including documents obtained in the theoretical sampling phase, were subjected to analysis using a combination of the constant comparative process, coding procedures, and interpretation.

Data collection continued until sufficient data was generated to allow for a comprehensive exploration of the research area.

### Analytical procedure

Initial or open coding in this research involved analysis of the interview transcription incident by incident or line by line, where each segment of meaningful data was named or coded. Initial coding was the first step in the analysis process, which was conducted by naming these segments of data or unit of meaning, using significant words in the data (Charmaz, 2014).

During open coding, the researcher was engaged in the process of interpretation and sought to interpret the meaning of each segment of data. This process is important in attending to participant voices, participant points of view, and interpretation (Strauss & Corbin, 1998a). This process conformed to the methods of Charmaz (2009) which acknowledge the possible existence of multiple perspectives in the process of meaning production.

Once the initial coding procedure was completed, sets of codes were constructed. These provided insight into the type of data required for the next interview. The process of initial coding procedures involved interpretation of units of meaning, constant comparison, theoretical sensitivity, and memo writing. This initial coding procedure was conducted for each of the 10 interview transcripts. Upon the completion of the coding procedure of the 10th transcript, 10 sets of coded data with 1316 codes were generated. The initial codes were then developed at a more conceptual level through focused coding.

Focused coding was initiated following the generation of codes produced from the 10 initial interview transcripts. Focused coding is more selective and conceptual (Glaser, 1978). In this research, focused coding involved grouping significant sets of codes that explained nearly similar concepts, to shift the data to the abstract level. This process was achieved by making decisions about the most significant codes as those that made the most analytic sense (Charmaz, 2014). Those codes that had conceptual power were selected, then conceptually categorized into codes that explained what was happening in the data (Glaser, 1978, p. 56).

Eight focused codes were identified from the data:

1. The arbitrary scope of practice
2. If we know what to do
3. It seems we can do anything, yet we have no authority
4. We learn while we work
5. Changing scope of practice
6. Learning from past experience
7. Fighting for recognition
8. Informal process of learning.

Through focused coding, eight codes were generated from the 1316 initial codes produced from the 10 initial interview transcripts. Furthermore, data generated from *the second round* of interviews and other data were incorporated into the process of focused code development. The researcher engaged in the categorization of all codes that had similar concepts. Throughout this analytical process, categorization of all codes that had similar concepts was undertaken. Memo writing was conducted to record ideas, information, and concepts that emerged in this focused coding procedure. Thus, eight focused codes were identified in this process. An example of the focused coding process is reflected in Figure 1.

**Figure 2.**
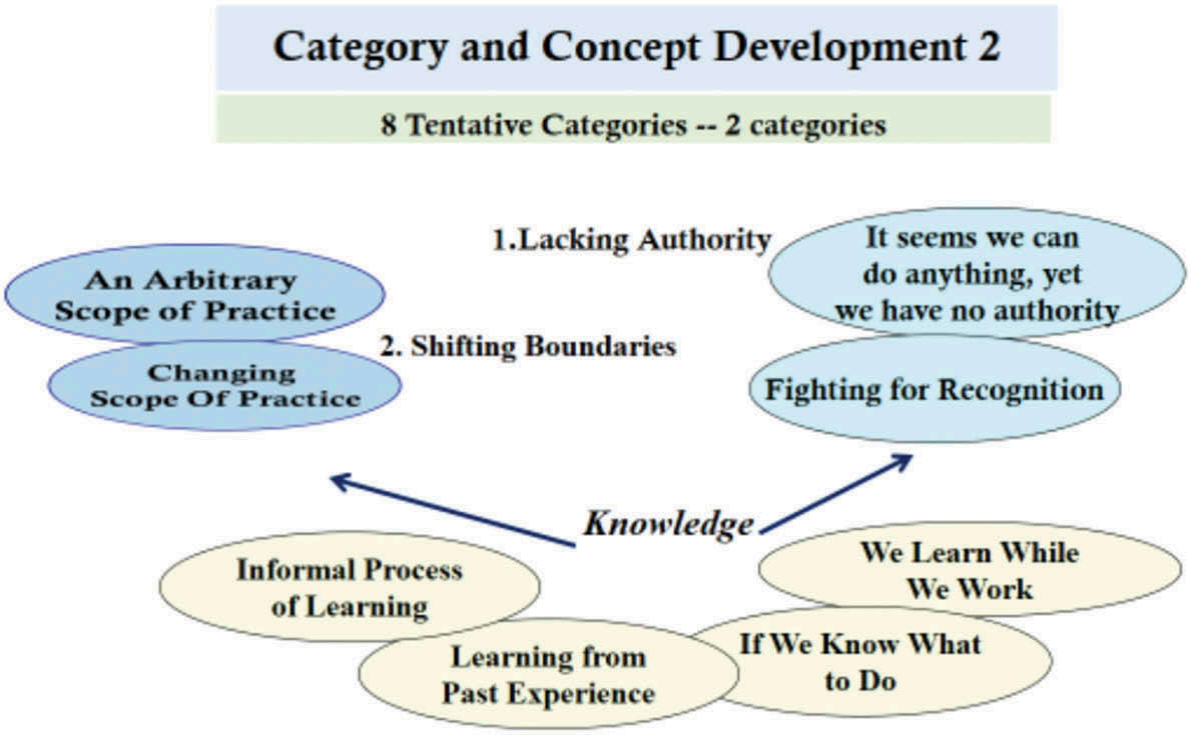
Concepts development.

In the concept development process, those eight focused codes were raised to a higher level of abstraction in the form of tentative concepts or categories. The tentative concepts were evaluated against all data and also all other tentative concepts. The most analytically significant concepts were determined where they demonstrated abstract power, expressed a general meaning, and showed analytical direction (Charmaz, 2014). The concepts development process is illustrated in Figure 2.

### Criteria for assessing the rigour of this research

The criteria for judging the quality of this GT study were drawn from the Glaser (1967) concepts of work, relevance, and modifiability. The concept “work” ensured that the theoretical explanation resulting from the research explained the phenomenon under research; in this research it was maintained by implementing the GT method from Charmaz (2009, 2014), which is grounded in pragmatism and social constructionism.

“Relevance” refers to the degree to which the theory addresses issues of importance to the research in question. It is concerned with the relevance of the core category to the data, and the other concepts (Hall & Callery, 2001). Relevance has been achieved by establishing a research question that originated from a knowledge gap, revealed in the field of practice. Relevance was also secured by ensuring conformity between the research question, the methodology, and the theoretical perspectives that provided guidance in the conduct of this research.

“Modifiability” refers to the core category in terms of its relevance, its workability, and its propensity for qualification and modification, leading to the enhanced density that can accurately reflect the phenomenon under research (Glaser, 1978). Modifiability was assured through the accomplishment of relevance and workability, which ensured that the theoretical explanations produced by this research accurately depicted the phenomenon under research.

## Research findings

The two key concepts generated in the research were *lacking authority* and *shifting practice boundaries* which reflected the constituent conceptual parts that constructed the core category of securing legitimate power.

### Lacking authority

The concept, *lacking authority*, reflects one dimension of the social process that constructed the role of the ED nurses within the Indonesian health care system. The nursing roles were constructed by complex social processes. 

Conceptually, authority has been explained as having control or power related to particular work, as well as control over the financial reward for such work. Authority is also understood as a type of social relationship among actors whereby authority holders automatically enjoy legitimacy (Peterson, 2012).

*Lacking authority* in the ED research context was manifest in the expansion of the scope of practice of the ED role. It was argued above that, as is the case in many countries, the expansion of nursing roles in emergency care settings has been a response to insufficient number of nurses and doctors appearing in health care services (Currie & Watterson, 2009). Thus role expansion within nursing is far more often an organizational imperative than the result of any decisions made by nurses.

In this context, the lack of authority of ED nurses was shaped by the intersection of historical, socio-cultural, political, and gender factors which saw the marginalization of nursing from university education.

Historically and culturally, the perception of women as subservient, docile appendages of husbands had been formative in Indonesia (Suryakusuma, 1996). The social positioning of women has informed views of the nursing profession whereby it is perceived that nursing work does not require higher-degree education. This converges with the findings of Ford and Parker (2008), who argue that women have been marginalized because of their unequal access to political involvemen in the development processes of new workforces.

The social-cultural and historical legacy grounded in gender issues underpins the view of nursing as women’s work. The social construction of nursing as women’s work has also resulted in the Indonesian nursing community commanding less political power at the health policy level. An example is the fact that Indonesian nursing was unable to secure professional status and the regulation of nursing for 25 years.

Professional regulation bestows authority through a process of exclusionary closure where legal boundaries separate one occupation from another (Saks, 2010). The lack of authority of nursing in the clinical setting, therefore, is grounded in the delay of Indonesian nursing to gain professional regulation for several decades. This social process means the entrenchment of inequality in authority at the workplace and a lack of recognition for nursing work in ED. The importance of authority understood in these terms is explained by one participant:

“The issue of authority for the conduct of advanced practice in the ED setting is a very important factor in the daily practice of nurses. This is because authority serves as a reference for nursing practice. Unclear authority means not knowing what to do and what not to do. Overt authority is critical for nurses, as practising without authority can potentially put nurses at risk” (CC).

It has been argued above that authority is reflected in the level of control nurses exercise over nursing work that is grounded in the legitimacy or legal status that originates from regulations around to nursing practice. This data converges with the work of Haug (1998), who argued that law and regulation served as one source of authority. In the health care system, knowledge and expertise are also recognized as a source of authority or power (Freidson, 1988a; Haug, 1998). This indicates that nursing authority is also mediated through policy at the national level.

The concept of authority in the current research reflected the degree to which the ED nurses controlled (or did not control) their practice by virtue of the positioning of nurses within the Indonesian health care structure. The level of nurses’ authority was shaped, in turn, by historical, political, and social processes.

The transition of Indonesian nursing from the vocational to professional level has thus been prolonged. The nursing work undertaken by trained personnel has existed since 1912. However, it was 49 years later, in 1961 that Indonesian nurses gained access to a *diploma 3* nursing education and it was not until 73 years later university-based nursing education was initiated at that University of Indonesia, in 1985. This social-cultural and historical legacy means that the majority of clinical nurses have not had access to university education. This situation can also be observed in the ED setting, where most of the nurses had a formal level education at *diploma 3*.

In this research, the participants were of the view that they required formal education to support their role and to ensure consistency of practice in the ED setting, as can be seen below:

“Of the 39 nurses who work in this ED, around 70% have experience in performing life-saving…. However, none are prepared formally (have formal education) to work in the emergency setting. Also, only five nurses have nursing educational backgrounds on BSN degree” (MNS, 2014).

In this context, delayed professionalization of nursing had resulted in inequalities in formal education levels, and sustained medical doctors in positions synonymous with power and as authority holders. For ED nurses, inequality in formal knowledge means inequitable levels of authority.

Formal knowledge at an advanced level, as indicated by Freidson (1988a), is a source of authority for professionals, since such expert knowledge and expert workers have the capacity to establish authority for their discipline and to create, preserve, transmit, assess, and revise the knowledge system of the discipline (Freidson, 2001). Knowledge was assumed as the basis of professional power and as guarantor of professional authority both of which are protected by professional autonomy (Coburn, 2006).

Authority, in the research context, was thus a condition linked social- historically to the position of nursing as women’s work, which was further sustained by the altruistic values that prevailed within the Indonesian nursing community. Ultimately, prevailing social values ensured the continued subordination of nursing within the Indonesia health care structure.

“Nursing work varies from providing information to patient families … to basic nursing interventions and to advanced interventions. The nurses (senior nurses) in the ED are also required to perform tasks such as life-saving interventions…. This is so even though, in reality, such practices are seen as a medical responsibility and are informally delegated to ED nurses” (OMN).

At the clinical practice level, nursing roles had been extended and yet without attracting the associated authority and the result was the entrenchment of inequality in the workplace and a lack of recognition for nursing work.

Furthermore, the notion of nursing as altruistic has sustained the subordination of nursing. The notion of altruism lends legitimacy to the context of “lacking authority” and inadequate formal education experienced by nurses. This can be seen in a research participant’s statement below:

“My first priority is to help the patient, and I will do what I can do and even with only verbal approval from the doctors, I (will do) life-saving intervention. Our first priority is to provide health services as soon as possible, to help the patient who needs the service. After all, if nurses don’t do this, who will?” (AD, 2009).

ED nurses self-define their role as taking action to save the lives of other people as a priority at the front line in emergency care services. The image of nursing as an altruistic profession has also resulted in a disconnect of nursing from a higher level of knowledge. The complex skills, expertise, and experience that require formal education at an advanced level for nurses have been inadequately recognized by society (Gordon & Nelson, 2005).

The extension of nursing work beyond traditional boundaries was unavoidable, as ED nurses were positioned at the forefront of the emergency care services. Because of their experience, senior nurses were capable of dealing with the full range of cases and interventions in EDs. The condition, *lacking authority*, reflects the fact that much of the extended activity of the ED nurses was formally and legally recognized as medical work, as evident in the following:

“Life-saving interventions or advanced interventions such as suturing … are conducted by nurses. There are a limited number of doctors” (AD).

A further participant noted:

“We do external fixation and cleansing of wounds and manage critical patient conditions caused by trauma.… We do an artery ligature first before sending them to the operating room … this requires special skill. Special skills are needed to manage patients with an open abdominal trauma. Also, if a patient’s condition is getting worse, turning into pre-shock, we report to the ED doctor…” (EC).

The invisibility of emergency nursing knowledge served to reinforce the dominance of medical knowledge. Indeed, the extended work undertaken by nurses was socially and politically constructed as knowledge belonging to the medical sector.

An associated lack of authority also meant an absence of formal recognition for any expansion of the nursing role in the clinical setting, as can be seen in the research participant comment below:

“The life-saving procedures we have responsibility for are less recognized. This is because all advanced life-saving interventions are recognized as medical interventions, so the allocation of financial rewards is mostly for authority holder….” (MO).

Advanced life-saving interventions are recognized as the work of the authority holder, as formally advanced practice nursing is still not recognized. For ED nursing at the micro level, formal knowledge at the advanced level is one source of authority and also formal and social recognition in the workplace. For a profession, specialized knowledge and self-regulation are the primary sources of authority.

Weber (1947) early on, and more recently Coburn (2006), argued that law and self-regulation were integral sources of authority. Freidson (1988b), Haug (1998), Zimmerman and Zeitz (2002), and Brante (2010) have also asserted that expert knowledge is a source of authority in the health care context. Professional authority is derived from that which is defined as professional expertise that is linked to expert knowledge, which is shaped by historical, political, and social processes relevant to nursing.

Delays in achieving professional status for advanced practice in Indonesian nursing is reflected in inequities in access to higher education and in a scarcity of authority. For Indonesian nursing, the social perception of nursing as women’s work has also created less political power at the health policy level. Wherever nursing has been perceived as women’s work, the voice of the nursing community has been granted little legitimacy or respect in policy debates (Davies, 1995).

Inequalities in formal education have created inequitable authority and lack of formal recognition for the extended ED nursing work. The lack of legitimacy afforded nursing knowledge underpinned the social and political positioning of the ED nurses. The structural and interactional features of ED nursing work, as a consequence, meant that nurses had little capacity to negotiate work boundaries.

### Shifting boundaries

Work on occupational boundaries tends to infer the stability of professions. The concept of boundary work relates to professional autonomy, authority and expertise (Gieryn, 1983), to the field of practice and professional knowledge (Fournier, 2000). In this research, the phenomenon of shifting boundaries explains the interrelationship between the ED nursing role and the wider social system within which nursing work has been situated. Work boundaries are constantly contested and continually evolving (Nancarrow & Borthwick, 2005).

The conceptualization of shifting work boundaries was perceived in terms of the extension of the ED nurses’ clinical activities. As noted below, where nursing work was extended, nurses redefined their work boundaries and consequently the boundaries of medical practice in general:

“Interventions in the ED … are mostly conducted by nurses, either independent nursing interventions or delegated interventions including life-saving. Mostly, ED doctors focus on providing medical therapy to other patients in this ED” (MN).

The participants perceived that where the nursing work in the ED was extended, the boundaries of their daily practice are blurred.

A blurring of work boundaries between health professionals is considered inevitable (Allen & Lyne, 1997; Nancarrow & Borthwick, 2005), and yet will take varied forms within different contexts. For the Indonesian ED nursing role, flexible work boundaries meant a shift in the demarcation of nursing work beyond traditional borders in response to internal and external factors. Internal factors, as noted above, were interrelationships within the health care context. External factors were the increasing demand for ED care services, a shortage of ED medical and nursing staff, and the public’s rising expectations in the quality of health care services, all of which have shifted demarcation of nursing work and manifested as the extension of the ED nursing role.

The broadening of the ED nursing role has occurred globally, as reported by Neades (1997), Hoskins (2011), Hodge, Perry, Daly, Hagness, and Tracy (2011), and Niezen and Mathijssen (2014). What differs across contexts is how socio-cultural and political factors constructed the extended nursing role

Integral to the phenomenon of shifting boundaries is the process of role substitution. For the ED nurses, role substitution signified “an extension of work activity” without legal definition, as the following research participant explains:

“So, if the doctor is not available, the doctor in triage will delegate (verbally) the advanced interventions to senior nurses …” (TN).

The process of vertical role substitution is relevant to the ED nursing role in Indonesia, where informal delegation of work occurs regardless of qualifications. Thus, flexible work boundaries exist along with role substitution, where a lack of equivalency of education, power, and authority facilitates the delegation of tasks across professional boundaries (Nancarrow, 2004).

Task and role substitution within the Indonesian ED nursing sector implied a need for higher education to ensure that nurses had the capacity to meet the challenges of an expanded nursing role. The link between the expanded ED nursing work and the need for higher education is indicated by Hoskins (2011).

The phenomenon of vertical role substitution was a complex phenomenon in the research context and somewhat contradictory. On the one hand, a changing scope of practice was indicative of extended ED nursing work and even, if without formal recognition, of an advanced nursing role. On the other hand, the blurring of boundaries had created a situation where emergency nursing as a specialized field remained largely invisible.

In this context, the extended nursing role was unrelated to education qualifications which would have underpinned authority over that expanded role. The blurred boundaries of nursing work in the ED setting are attributable partly to the informal learning process that ED nurses must engage to acquire the requisite knowledge and skills. Informal learning, although critical to the ED nurses in the research, produced nursing knowledge and skills that were largely tacit and hidden. Indeed, informal learning is associated with silent forms of knowledge (Eraut, 2004). This informal process of learning, in turn, produced emergency nursing knowledge that was largely invisible.

As discussed above, shifted practice boundaries in this context are not only related to professional knowledge and professional authority but also link to the absence of a legal framework for advanced practice nursing, in turn, meant the non-existence of legally-defined boundaries for expanded nursing work. The phenomenon of shifting boundaries has indicated the importance interrelationship between professional knowledge at advanced level, authority and power.

### Core category: securing legitimate power

**Figure 3.**
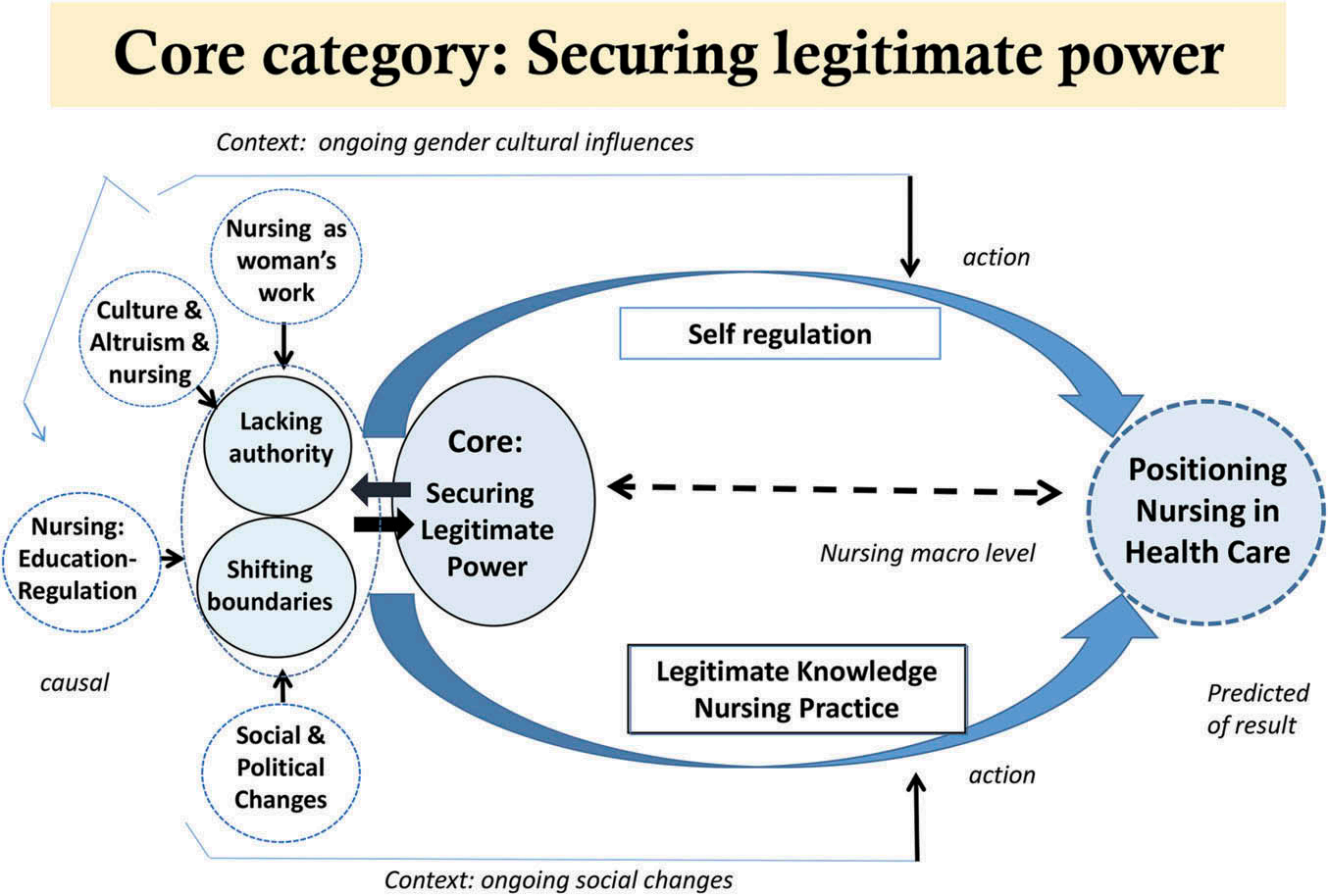
Core category: securing legitimate power.

The core category provides an understanding of theoretical relationships among concepts or categories. The inter-connection between nursing at the micro level and the point at which all constituent elements interrelated depicts the conditions of ED clinical nursing (as indicated in Figure 3). These conditions were characterized by lack of authority and shifting practice boundaries, the key concepts of this research.

S*ecuring legitimate power* was the basic social structural process that reflected the evolution of nursing in the Indonesian ED and it is relevant to the evolution of nursing worldwide.

As depicted by the categories, the core category represents the interrelationships between self-regulation, legitimate knowledge and practice. These concepts were central to the positioning of the nursing profession, and the ED nurses’ role, within Indonesian health care.

As is the case elsewhere, in Indonesia, the research phenomenon was embedded in the wider society. Within that context, culture, gender and power relations prevailed as constraints on Indonesian nursing. Nonetheless and reflecting Mead’s (1934) work, the participant ED nurses were active meaning creators in Indonesian nursing. As such, we start with the assumption that the position of the ED nurses in this research is understood as the interrelationship between social context and action.

The point at which all constituent elements of the research phenomenon interrelated depicts the conditions of ED clinical nursing in Indonesia. The evolution of nursing globally has been embedded in an inequitable distribution of authority. In this research, lack of authority and shifting practice boundaries were indicative of the interplay between power (professional autonomy/regulation), knowledge, culture and gender that gave rise to conflict. Yet, conflict was an essential part of the process of change in Indonesian nursing. Conflict provokes innovation and creativity and also new institutions that might lead to the improvement of a social system (Coser, 1957). While social change had demanded an expansion of the ED nursing role, without conflict, recognition of the legitimacy of the changed scope of practice would remain subdued.

## Discussion

The contextual conditions that shaped the ED nursing role cannot be argued in isolation from gender and culture. Indonesia, in general, is characterized by a strongly patriarchal society (Clark, 2010) that has produced and reinforced the subordination of Indonesian women. Yet, culture is also always in the process of “change” and is “emerging and evolving” as a consequence of social, political and other changes (Wyer, Chiu, & Hong, 2013, p. 45). As a system of values and a repository of shared knowledge (Geertz, 1973) that is communicated and evolves continually, culture cannot be separated from its interplay with power.

### Gender and professionalization

The delay in professionalization of Indonesian nursing is linked to gender and cultural issues. Professionalization is understood as a mechanism for occupational advancement to be achieved through internal methods of self-regulation (Evetts, 2003, 2013). 

The interaction of gender and the health care system in Indonesia reflects what Meerabeau (2005) refers to as a gendered substructure of organization. The politics around occupational boundaries were also gendered in this research where a male dominated profession sustained control through the mechanism of delegation. For *advanced practice nursing* this interaction means the importance of links between *advanced practice nursing* with advanced knowledge and skills at higher education and political power including professional autonomy.

### Self- regulation

As in many countries, in Indonesia, the institution of professional self-regulation was associated with a sharing of power, in the governance of nursing between the *State* and the nursing profession. The recognition of a profession starts with the achievement of professional self-regulation or professional autonomy which indicates that a government has granted a body the status of a fully-fledged profession (Saks, 2010). Led by INNA, the Indonesian nursing society had been involved in political and social action over 25 years. Nursing law No. 38, 2014 (The Republic of Indonesia, 2014) was approved in September 2014. This law provided a legal basis for self-regulation and to shape nursing practice (Adams, 2009).

In Indonesia context, growing political support for nursing self-governance coincided with a rise in the number of nurses with higher degrees and the growth of higher degree nursing institutions in Indonesia. For nursing, in Indonesia and many countries, both political processes and the existence of a body of professional knowledge are central to the achievement of professional self-regulation and professional autonomy.

### Legitimate knowledge and nursing practice

The securing of legitimate knowledge for ED nurses in Indonesia can be explained as a struggle to shift what is largely invisible nursing knowledge from the margins in health care to a position where that knowledge is recognized by the Indonesian health care system and the broader society. Legitimate knowledge is a dimension of a process that engenders within nursing the authority, formal recognition, and legitimate power that is critical for positioning nursing in the health care system. Legitimate knowledge is that which is communicated in an academic setting, is known as official knowledge and is generated and protected through professional or institutional research (Apple, 1992). However, in the health care context, the construction of legitimate knowledge has involved the struggle of occupations shaped by gender, the state, dominant professions, society, and formal knowledge at the higher degree level. In this case, expert knowledge is important for the process of both maintaining and expanding professional positions and for professionalization (Allsop & Saks, 2002).

As is the case globally, in Indonesia the construction of nursing knowledge as legitimate also requires broader structural change since the production of such knowledge is related to a gendered struggle for equality. The gendered exclusion of nursing in the production of legitimate knowledge has manifested as a disconnect between nursing and the nexus of knowledge at the higher degree level, and thus power or professional autonomy.

Kuhlmann (2006) indicated that the making of legitimate knowledge acknowledges the importance of structural change and the power of professional knowledge to reconstruct boundary work. Such structural change involves the equal participation of women in the regulatory processes and practices which is considered a prerequisite for the achievement of effective governance (United National Development Programme, 2014). It also includes the development of gender-mainstreaming policy in the health care system, in Indonesia and in all countries in the World.

## Conclusion

As is the case in many countries, the interconnection between political gains, tertiary knowledge, and professional regulation has been the focus of Indonesian nursing in gaining professional authority and positioning nursing more strongly within the health care system. Professional regulation is critical to professional power, as it engenders in nursing the right to self-governance. Self-governance, in turn, is just as much a condition for, rather than a product of, control over an area of knowledge. Similarly, the expansion of nursing education at the post graduate level, of itself, is more an endpoint to a long struggle for political strength to establish professional autonomy for Indonesian nursing.

The repositioning of nursing in the health care system in Indonesia and many countries also requires the political will of the government to develop gender-mainstreaming policies that reflect equal access and participation for nursing. Nursing cannot sit outside the dominant social structure that gives rise to a hierarchy of occupations and thus, legitimate knowledge is critical in claiming an area of expertise for nursing within the existing health care system.

## Limitations of the research

This GT study stresses the importance of reviewing literature in the Indonesian context. However, there were no prior studies related to the nursing role in the Indonesian context. Prior understanding of the research problem, therefore, was obtained largely through related literature from the international setting. The issues have been mediated by comparing and linking the research area to the conditions in Indonesian context.
